# Phenotyping sarcoidosis: a single institution retrospective analysis

**DOI:** 10.3389/fmed.2025.1590102

**Published:** 2025-05-07

**Authors:** Francesco Rocco Bertuccio, Davide Piloni, Marianna Russo, Fady Tousa, Mariachiara Crescenzi, Paola Putignano, Nicola Baio, Ida Maragò, Angelo Guido Corsico, Giulia Maria Stella

**Affiliations:** ^1^Cardiothoracic and Vascular Department, Unit of Respiratory Disease, IRCCS Policlinico San Matteo, Pavia, Italy; ^2^Department of Internal Medicine and Pharmacology, University of Pavia, Pavia, Italy; ^3^Department of Internal Medicine and Medical Therapeutics, University of Pavia Medical School, Pavia, Italy

**Keywords:** sarcoidosis, phenotyping, interstitial lung disease, inflammatory granulomatous diseases, granulomatous diseases

## Abstract

**Introduction:**

Sarcoidosis is a systemic disorder marked by the presence of non-caseating epithelioid cell granulomas. The diagnosis relies on a consistent clinical presentation, histological evidence of non-necrotizing granulomatous inflammation in one or more tissue specimens, and the exclusion of other potential etiologies of granulomatous disease. It is a heterogeneous disease with many focal points to be clarified. For instance, finding a relationship between symptom burden, race, gender, HRQoL, and pulmonary function could have therapeutic ramifications, influence clinical practice, and aid in selecting patients for specific clinical studies.

**Methods:**

A comprehensive statistical evaluation was conducted using the JMP partitioning algorithm which explores all potential divisions to identify the most predictive variables.

**Results:**

With our analysis, we tried to categorize patients from a single Institution respiratory unit to delineate clinical phenotypes in sarcoidosis.

**Conclusions:**

Larger studies using appropriate methodology should surely be carried out to address this issue and help clarify the varying contributions of genetics, socioeconomic status, environmental exposures, and other sociodemographic factors to illness severity and phenotypic presentation. Additionally, the application of transcriptomics, interdisciplinary methods, patients' disease perspectives, and the publishing of novel discoveries may contribute to enhanced clinical support and a deeper comprehension of the etiology of illness.

## Introduction

Sarcoidosis is a systemic disorder marked by the presence of non-caseating epithelioid cell granulomas. The diagnosis relies on a consistent clinical presentation, histological evidence of non-necrotizing granulomatous inflammation in one or more tissue specimens, and the exclusion of other potential etiologies of granulomatous disease (1, 2).

Over 50% of cases of sarcoidosis occur in persons over 50 years of age, with a small female predominance (female: male ratio 1.2 to 1.5:1) and an estimated incidence of 1 to 40 cases per 100,000 people (3, 4). The lungs, lymph nodes, skin, and eyes are the organs most frequently affected (1, 5). Even though sarcoidosis has a very unpredictable natural history, up to two-thirds of patients may have spontaneous remission; some cases, however, may experience a chronic and progressive disease course or even manifest symptoms that could be fatal (5). Because of this clinical variety, it became necessary to identify groups of patients (sarcoidosis phenotypes) who presented with comparable clinical patterns and prognoses (6).

The use of biomarkers, PET-CT, and CT pattern scores has improved organ involvement evaluation and diagnosis. Because pulmonary sarcoidosis spontaneously remits at a specific rate, some individuals do not need systemic treatment. Based on the clinical presentation, the organs impacted, the extent of disease, and any underlying medical conditions, a tailored treatment plan must be planned.

The following conditions fall under the guidelines for treatment: involvement of extrapulmonary sites such as the cardiovascular, dermatological, or nervous systems; pulmonary disease with respiratory symptoms and/or functional impairment; and disease-related loss of quality of life. At the moment, the first line of treatment for sarcoidosis is based on corticosteroids; second line on cytotoxic drugs and the third line on anti-tumor necrosis factor (TNF) biologics. Novel treatments for sarcoidosis, including as rituximab and repository corticotropin injection, have been studied with promising results (1, 2).

The multidisciplinary ERS task force created eight clinical questions in 2021 that focused on treating sarcoidosis using the PICO (Patients, Intervention, Comparison, Outcomes) style. The recommendations were based on the GRADE (Grading of Recommendations Assessment, Development and Evaluation) methodology. The committee examined the management of related tiredness and small fiber neuropathy as well as respiratory, cutaneous, cardiac, and neurologic symptoms. Nevertheless, no particular recommendations about dosage, supervision, or length of treatment for any kind of illness were provided (2, 7).

## Phenotyping

Owing to the clinical variability of sarcoidosis, numerous research endeavors have attempted to identify phenotypic subgroups that may be able to forecast a patient's outcome and, in turn, assist medical professionals in selecting a particular therapeutic approach and/or the necessity of referring patients to highly specialized healthcare facilities (8–10). The first effort at a phenotypical staging of sarcoidosis was made in 1960 by Karl Wurm (11) and in 1961 by Guy Scadding (12). The results of the chest radiograph, which demonstrated the involvement of the lungs and hilar lymph nodes, were the only basis for this classification. However, research on the connection between the Scadding categorization and the sarcoidosis phenotype has yielded contradictory results (6, 12).

In 1961, Scadding discovered that certain additional thoracic features had no connection with radiological appearance. Moreover, research showed a limited correlation with the need for medication, pulmonary function tests, or the severity of the condition (11, 13–16).

On the other hand, several studies have found a favorable link with the prognosis, diagnosis delay, bronchial granuloma density, lung volumes, respiratory symptoms, CT-scan scores, and gender (8–10, 12, 17, 18).

There are several approaches to identifying clinical phenotypes. Utilizing clinical, physiological, and radiologic data to identify variables that impact disease heterogeneity and may therefore be pertinent to diagnosis, prognosis, or both is one method of phenotyping (19). Any clinical phenotype must, by definition, be associated with a minimum of one clinically significant outcome. This inherently suggests that prospective validation is necessary for any clinical characteristic.

Using analytical or multivariate tools to describe phenotypes based on various features of an illness is another option. It does this by including a wider range of observable attributes, which raises the possibility of finding meaningful relationships. Second, rather than focusing on the exact manifestation of a particular illness feature, it analyzes the relationships between the several disease domains to describe each phenotype. Stated differently, analyzing the connections among domains is probably going to help define the underlying mechanisms and routes more precisely.

The study of phenotypes has benefited greatly from the application of analytical techniques, which have made it easier to identify distinctive groups made up of various variables to find potential relationships between endotypes—the underlying biology of disease—and clinically meaningful outcomes. These methods, which are usually hypothesis-independent, find correlations between clinical factors and outcomes by utilizing data from sizable cohorts of thoroughly defined patients.

Perez-Alvarez et al. analyzed ~1,230 patients in 2019 to look for a systemic phenotype of sarcoidosis linked to radiological phases. The authors discovered that in patients with sarcoidosis from Apart from a higher chance of concomitant abdominal involvement (liver, spleen), pulmonary involvement as shown by the Scadding stages was associated with a distinct systemic phenotype at diagnosis in Southern Europe. Patients without lung involvement, on the other hand, were more likely to experience systemic disease overall, with a particular increased risk of simultaneous cutaneous/musculoskeletal features (Löfgren's phenotype) and cephalic extra thoracic involvements (ENT, ophthalmic, neurological). It is a useful approach in all kinds of clinical settings (especially primary care), as these correlation patterns may help physicians who suspect an extra thoracic involvement during the diagnostic work-up based on the thoracic organs implicated in the chest X-ray. These findings suggested that thoracic organ analysis alone, as opposed to patient clustering based on individual Scadding stages, improves sarcoidosis phenotyping since the latter includes organs whose involvement varies across stages and has contradictory prognoses, thereby requiring contradictory therapeutic approaches (20).

The creation of an agreement on the management and therapy of pulmonary sarcoidosis has been hampered by the lack of success in attempts to develop a reliable prognostic algorithm, even though several studies have identified factors (such as reduced forced vital capacity, pulmonary arterial hypertension, and the radiographic presence of pulmonary fibrosis) that may predict prognosis in pulmonary sarcoidosis (11, 13, 14, 21).

Walsh et al. (22) proposed a staging system that identifies pulmonary sarcoidosis patients at high clinical risk. This staging approach was developed using the composite physiological index and two HRCT variables from a large patient cohort with pulmonary sarcoidosis. Since most patients have these tests regularly, the suggested staging method can be used for individuals in less selective populations who have pulmonary sarcoidosis. Ideally, this staging approach should be evaluated further in less specialized centers or patients with less severe disease.

The primary outcome was mortality. They calculated the survival time from the date of the baseline pulmonary function tests to the date of death or, for survivors, the last known point of contact. All patients were aware of their vital condition following the experiment period.

This model has the potential to serve as an effective yet straightforward predictive tool for patient risk stratification in standard clinical practice and clinical trial enrolment (22).

The largest and most recent attempt at phenotypic categorization was published in 2020 by Baughman et al. in the form of an Expert Delphi consensus recommendation on clinical phenotyping to guide therapy for pulmonary sarcoidosis. The panelists concurred that asymptomatic patients with normal pulmonary function, adenopathy alone, or normal chest imaging do not require medication, but symptomatic patients with compromised pulmonary function or infiltrates should receive treatment.

The panel was unable to reach a consensus on whether asymptomatic patients had abnormal chest imaging and reduced pulmonary function, or symptomatic patients had normal chest imaging and pulmonary function. The proposed phenotypic classifications and associated treatment recommendations are as follows: asymptomatic (no therapy), acute (disease duration < 1 year, likely self-limited, corticosteroids), chronic (antimetabolites and other second-line medications), and advanced (biologics).

Certain clinical conditions, like resting dyspnea or hypoxemia, quickly declining or significantly impaired pulmonary function tests, and significant involvement of the heart, brain, eyes, or kidneys, call for emergency treatment (23).

Certainly, analyzing risk factors could be a relevant point to evaluate.

A study on obesity and smoking as sarcoidosis risk factors was released in 2016. The authors came to the conclusion that these factors might influence the risk of sarcoidosis despite the ambiguous evidence (24). Lung cancer and chronic obstructive pulmonary disease are two conditions for which smoking is a significant risk factor. Interestingly, yet, earlier research has shown that smoking was linked to a lower incidence of sarcoidosis; nonetheless, those studies were carried out with referral-based cohorts, which may not accurately reflect the full range of the illness (15, 25). Psoriasis and rheumatoid arthritis are two autoimmune diseases that are more common in obese people (26, 27). A recent study using the Black Women's Health Study cohort found that African-American women with a body mass index (BMI) of greater than 30 kg/m2 are at higher risk for sarcoidosis (6). Information about the connection between obesity and sarcoidosis risk in other populations is currently lacking, though (28).

There is a direct correlation between the risk of sarcoidosis and current smoking. The outcomes matched those of earlier referral-based research conducted in the West. Why current smokers are less likely to develop sarcoidosis is unknown. It is well established that smoking reduces the function of T lymphocytes and the ability of macrophages to phagocytose. Consequently, smoking may potentially obstruct the process of macrophage-lymphocyte activation, which leads to the creation of granulomas (15, 16).

It has also been shown that smoking increases the incidence of hypersensitivity pneumonitis, another granulomatous inflammatory lung condition (29, 30). However, it should be mentioned that Asian cohorts have not shown a negative correlation between smoking and sarcoidosis (31, 32). Several etiopathogeneses probably cause sarcoidosis in various geographic areas or populations. Conversely, this study showed a positive correlation between the risk of sarcoidosis and obesity. Those who were overweight did not show the association. It is unknown what precise mechanism underlies the link between obesity and sarcoidosis. One probable cause is that obese patients' adipocytes secrete too much leptin. Sarcoidosis and autoimmunity are made more likely by leptin, a pro-inflammatory adipokine with strong immunomodulatory effects that can maintain autoreactive cell growth (33, 34).

Recent insights into immune regulation have identified several molecular and cellular pathways potentially contributing to the onset and progression of sarcoidosis. Among these, vitamin D has gained attention for its immunomodulatory functions. It is known to suppress Th1 and Th17 responses while enhancing regulatory T-cell activity, thereby promoting immune tolerance. In sarcoidosis, vitamin D deficiency has been frequently observed and is thought to both reflect and contribute to the systemic inflammatory burden. Activated macrophages within granulomas express 1α-hydroxylase, which locally converts 25(OH)D into its active form, potentially leading to systemic depletion and altered immune regulation (35).

The role of the microbiome has also been increasingly recognized. Dysbiosis, particularly at the pulmonary and intestinal mucosal interfaces, can promote aberrant immune activation by increasing epithelial permeability, altering antigen presentation, and affecting cytokine profiles. These microbial perturbations have been linked to chronic inflammatory and granulomatous conditions, including sarcoidosis. Since metabolites like short-chain fatty acids that are produced by the gut microbiota have a direct impact on lung immunity, the gut–lung axis seems to be at the heart of this process (36).

Importantly, a bidirectional relationship between vitamin D and the microbiome has been demonstrated. Vitamin D regulates epithelial barrier function and the expression of antimicrobial peptides, such as cathelicidin, which shape microbial communities and prevent dysbiosis. Conversely, microbiota composition can influence vitamin D metabolism and signaling pathways, thereby modulating systemic immunity. This interconnection may establish a feedback loop that promotes or attenuates inflammation in immune-mediated diseases like sarcoidosis (36).

From a cellular immunology perspective, Th17 cells are considered pivotal in sustaining granulomatous inflammation. Their hallmark cytokine, IL-17, promotes neutrophil recruitment and fibrotic remodeling. Th17-driven responses have been associated with disease chronicity and may interact with environmental and host genetic factors to shape clinical phenotypes (37).

The IL-31/IL-33 axis is another emerging player in chronic immune responses. IL-33 acts as an alarmin released upon tissue injury, amplifying type 2 immunity and facilitating epithelial–mesenchymal crosstalk. Its expression has been linked to tissue remodeling and symptom burden in inflammatory lung diseases, and its possible involvement in sarcoidosis warrants further (38).

Lastly, the contribution of non-antigen-specific CD8?/CD28? T suppressor cells to immune regulation is of growing interest. These cells exert suppressive effects on effector T-cell proliferation and cytokine production, maintaining immune homeostasis. Their dysfunction or numerical reduction has been implicated in several chronic immune-mediated diseases. In sarcoidosis, a failure in this suppressive mechanism could support prolonged immune activation and granuloma persistence (39).

These immunological pathways represent promising areas for translational research and may help identify novel biomarkers or therapeutic targets to better stratify patients and personalize treatment strategies.

### Influence of gender

Previous studies have demonstrated that there is variation between the sexes in terms of clinical phenotype and age distribution at diagnosis.

As an example, in 2022 Lundkvist et al. performed a study aimed to investigate whether there are differences in clinical presentation between Swedish men and women with sarcoidosis. Their cohort comprehended 1,429 patients and 61% were men.

Men were overrepresented in their local cohort, they were typically younger when the disease first manifested, and their radiographic stage on chest imaging was more advanced. Women's clinical presentations were marginally different, involving greater skin and salivary gland involvement.

Except for cutaneous involvement and salivary gland illness, there was no discernible difference in the frequency of involvement of each extra-pulmonary organ between men and women. Involvement of the skin was prevalent in the group. Even if EN (erythema nodosum) was excluded from the patient group with cutaneous involvement, skin involvement was nearly twice as likely in women. It has been suggested that women's sex hormones affect their chance of getting EN, which could account for a general inclination toward skin sarcoidosis. Additionally, it has been discovered that women report a greater degree of various symptoms and seek medical assistance more frequently than men (40).

In a different 2017 study, all Olmsted County, Minnesota, individuals who received a new sarcoidosis diagnosis between 1976 and 2013 were identified using data from the Rochester Epidemiology Project. The authors concluded that there was no variation in sex-specific susceptibility to sarcoidosis. In this study, females tended to have more uveitis and cutaneous involvement than males, and they also tended to develop sarcoidosis at an older age (41).

## Materials and methods

Moving from the above considerations our focus is based on the identification of risk factors for worse prognosis in our patients affected by sarcoidosis and to perform an attempt to phenotyping our population.

The purpose of our work was to review these concepts through a retrospective analysis involving 160 patients coming from the Respiratory Unit of IRCCS Policlinico San Matteo in the last 6 years in acute and outpatients settings.

For each patient entered into the database, we assessed several benchmarks:

- Age at diagnosis
- Sex
- Ethnicity
- Familiarity with chronic lung disease
- Occupational exposure
- Smoking
- BMI (Body Mass Index)
- Clinical-radiological and histological diagnosis
- Lymphocytosis in the cytogram
- Pulmonary hypertension
- Asthma
- Chronic bronchopneumopathy (COPD)
- Gastroesophageal reflux disease
- Connective tissue disorders
- Immunodeficiency
- Sleep disorders
- Cancer and its activity
- Arterial hypertension
- Heart disease
- Depression
- Anxiety
- Scadding Scale
- Imaging examinations such as CT and PET scans
- Respiratory function tests at diagnosis and after therapy
- Fatigue
- Myalgia
- Sputum
- Dyspnea at rest
- Cough
- Dyspnea evaluation test (mMRC)
- Haemopthysis
- Symptoms after therapy
- Anticholinergics and inhaled corticosteroids
- LTOT (Long Term Oxygen Therapy)
- Long-term noninvasive ventilation
- Corticosteroids
- Immunosuppressants
- Fibrosing therapy (Nintedanib)
- Duration of symptoms from the start of treatment
- Duration of therapy
- Relapses after treatment
- Quality of life with treatment and without treatment

### Data elaboration and analysis

A comprehensive statistical evaluation was conducted using the JMP partitioning algorithm (JMP—Statistical Discovery Software by SAS, available at http://www.jmp.com), which explores all potential divisions to identify the most predictive variables.

Decision trees represent a supervised classification technique, where a sequence of hierarchical decisions based on input variables is structured in a tree format. At each branching point, known as a split node, a decision rule is applied typically involving one or more predictor variables from the training dataset.

The dataset is recursively divided from the top of the tree downward, applying these rules in an iterative process. At each iteration, a subset of features is excluded based on the selected split criteria, and the model is subsequently retrained on the reduced set of variables to update the predictive weights.

This process resembles hierarchical clustering, though the key distinction lies in its supervision—the splits in decision trees are informed by class labels rather than relying solely on data structure.

This partitioning method is straightforward to implement, highly interpretable, and particularly valuable for developing diagnostic tools. Given a dataset comprising clinical features and outcomes, the algorithm can generate a sequence of diagnostic questions aimed at classifying new cases.

Predictive variables can be either continuous or categorical (nominal or ordinal). For continuous predictors, the algorithm determines a threshold value that divides the dataset into two groups, those below and those above this value. For categorical variables, the data is split into subgroups based on category levels.

Depending on the nature of the response variable, the platform estimates either the mean response (for continuous outcomes) or the probability distribution across response categories (for categorical outcomes).

To accurately assess classification uncertainty, several statistical measures are employed. The Gini index (IG), calculated from the relative proportions (pi) of the M response categories, reflects heterogeneity within a node. Another measure, entropy (H), quantifies the level of uncertainty or disorder (42).

These indices guide the algorithm in selecting the most appropriate split at each stage of the tree construction, ensuring an optimal partitioning strategy (19, 42).

## Results

Our analysis involved 160 patients (64 female and 96 male) with a mean age < 60 years. One hundred fifty-six are Caucasians, 3 Asians and 1 Afroamericans.

Airflow obstruction was identified at diagnosis in 14 patients while no restrictive pattern was registered.

Within respect to smoking habit 18 patients are current smokers and 39 former smokers. Notably, despite the significant number of patients with exposure to smoke in our cohort no patients showed a progressive fibrotic disease over time, consequently we have no patients currently in therapy with antifibrotic drug.

Overall, our data suggested that sarcoidosis could occur in smoker subjects, although it is more frequent among non-smokers. Moreover, fibrotic disease is associated with smoking exposure, while in out cohort despite about 1/3 of our patients had smoking exposure no fibrotic pattern was identified, consequently it would be relevant to investigate not only environmental risk factors, but also interindividual and gender characteristics.

Concerning with body mass index, 32 patients were obese and 43 overweight. A positive association between obesity and risk of sarcoidosis was demonstrated in 2016 as stated above while the association was not observed among subjects who were overweight in previous studies.

In almost every case diagnosis was confirmed histologically, except for 8 cases in which clinical-radiological diagnosis was possible (Lofgren and Heerfordt syndrome).

Endobronchial lung ultrasound lymph node biopsy was performed in 36 cases, video-assisted thoracoscopy in 12 patients. In the remaining case diagnosis was obtained by CT-scan fine needle aspiration, mediastinoscopy, superficial lymph node biopsy, transbronchial lung biopsy and Daniels' biopsy (scalene lymph nodes biopsy).

**Figure 1:**
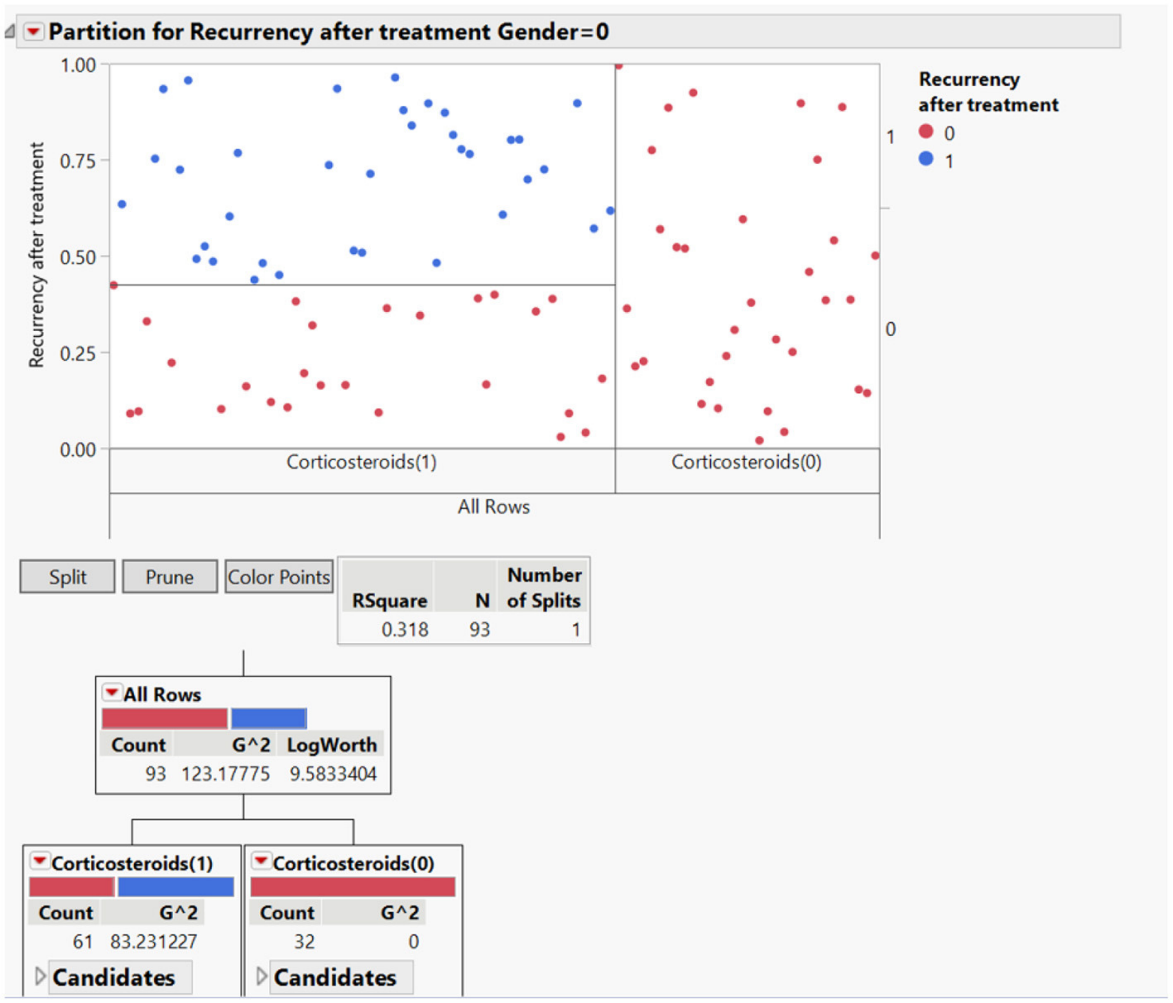
Partition for recurrency after treatment related with gender differences.

**Figure 2:**
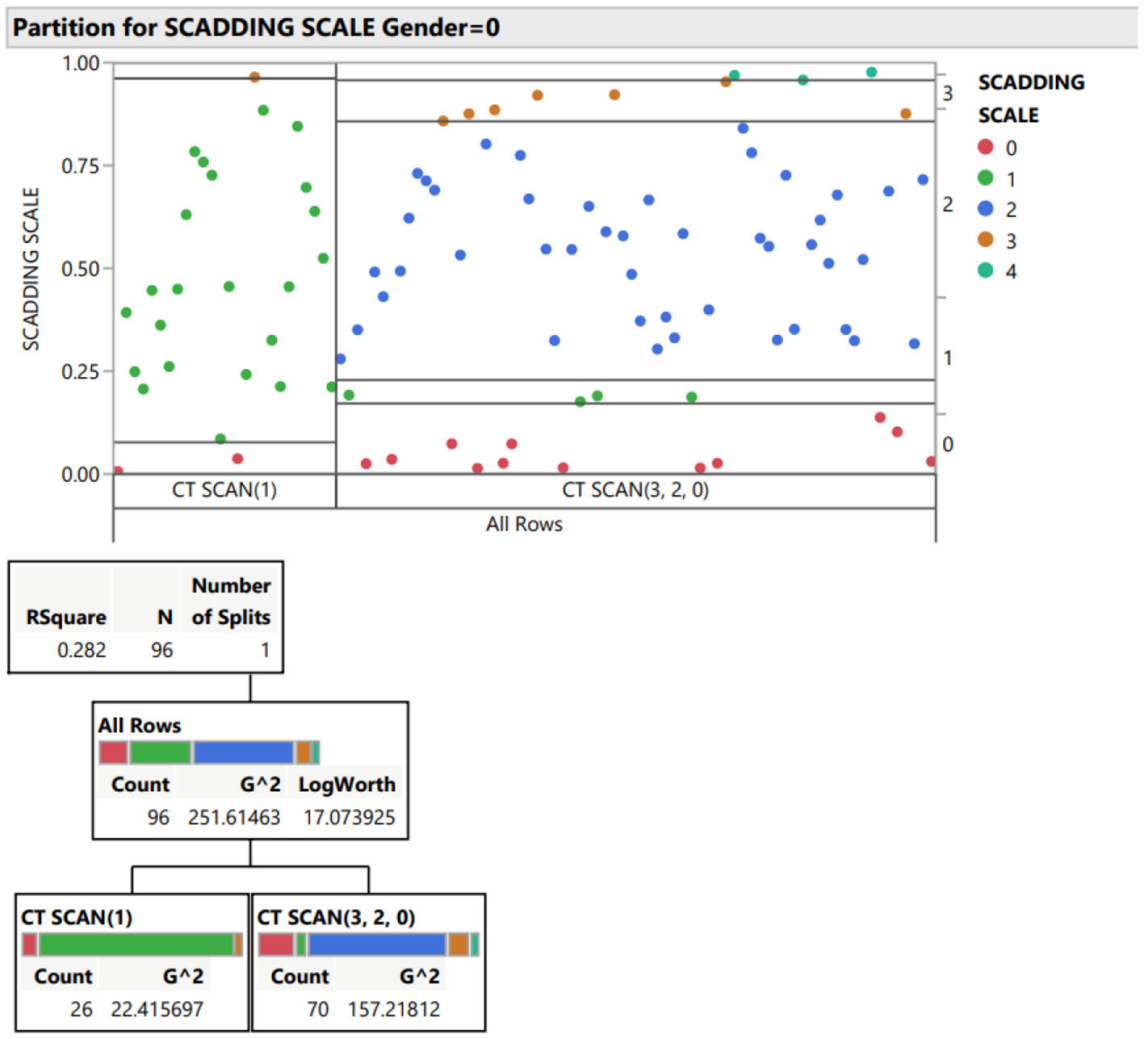
Partition for Scadding scale.

To clearly understand therapy-related relationship, we applied to the dataset a data mining suite for statistical analysis, through JMP program. This approach highlighted some unexpected findings: from our work it is clear that loss of quality of life is the most reported complaint by patients together with cough, weakness and dyspnea. Surprisingly, despite corticosteroid systemic therapy 20 patients reported persistence of symptoms and 61 recurrency of disease especially in stage 1 and 2 Scadding scale (Figures 1, 2).

Our data suggest that in early stages of disease without severe clinical symptoms it could be acceptable to have a “watchful waiting” approach before starting treatment. This concept is strengthened by the intolerance and drug-related side effects reported by 18 patients to systemic corticosteroids therapy.

It is commonly known that sex gender affects the clinical phenotype and epidemiology of a number of autoimmune diseases, including multiple sclerosis, ankylosing spondylitis, and systemic lupus erythematosus (20). There are, however, few data about the impact of gender on sarcoidosis. In this analysis we try to provide a more thorough description of the clinical illness manifestation and epidemiology of sarcoidosis in both male and female patients.

Concerning with smoking habits, in our cohort it is more common in men (34 previous smoker and 14 active smoker) than in women (6 active smoker and 10 previous smoker) and these data could be related to our obstructive disease findings, 11 in male and 4 in women.

As stated above, bodyweight can have an impact in sarcoidosis as in many autoimmune diseases. In our population, women obesity and overweight were higher than in men: 20 obese patients and 13 overweight out of 64 total women.

Out of the entire cohort, almost all (158) had lung involvement (i.e., Scadding stage I-IV on chest imaging). There was a significant difference between men and women with regards to the radiographic stage on chest imaging at disease onset, where more women had stage 2–3 disease on chest imaging (12 for stage 1, 27 for stage 2 and 11 for stage 3 out of 64 total patients). Men on the other hand presented with a less advanced radiographic stage on pulmonary scans, radiographic stage I-II (28 patients and 46, respectively); however, we identified jut 3 cases of fibrotic stage sarcoidosis stage 4 and they are all male (this data could be correlated to the more extensive smoking habits in our male population).

Pulmonary symptoms, including cough, dyspnea, and chest pain, were almost equally reported by man and women (dyspnea on effort and cough were the main reported symptoms in both sub-population).

The frequency of individual extra-thoracic organ involvement was higher in females than males (37 women out of 64), cutaneous involvement and uveitis were significantly more common among females while cardiac involvement (4 cases) were all men.

Dealing with treatment we find that recurrency of disease after treatment was significantly higher in men than in women: out of 56 men, 34 reported recurrency of symptoms after treatment. On the other hand, out of 48 women just 15 needed further treatment.

## Discussion

The clinical heterogeneity of sarcoidosis poses a challenge to the design of clinical trials and the selection of optimal outcomes, despite the fact that the development of medications that result in more effective and targeted treatment is necessary. The expert panel concluded that endpoints measuring wellbeing or inflammatory activity were clinically relevant, but they admitted that further endpoints might be required. It was also advised that quality of life (QOL) be measured in all research since QOL impairment is a regular occurrence and a factor in morbidity.

Phenotyping is becoming more and more helpful for clinical and scientific purposes as it advances our understanding of the disease. With this method, treatment can be more individually tailored, maximizing therapeutic efficacy and reducing side effects. Additionally, it makes it easier to create novel, focused diagnostic and treatment plans, which enhances the overall quality of life for sarcoidosis patients and helps to manage the condition better overall. Finding the genetic and environmental predispositions to sarcoidosis will also be crucial because these factors may be linked to increased mortality and more severe disease progression. Additionally, it might make it possible to create more focused treatment plans, which would enhance the disease's overall care.

Regarding the “wait and see” strategy, it is critical to conduct more thorough and frequent follow-up on patients in the early stages of sarcoidosis. Regular clinical, radiographic, and laboratory monitoring should be part of this to make sure that any worsening is identified and that, in the event that progression is evident, therapy is started right once to avoid consequences.

One of the primary limitations of this study is its single-center design, which inherently restricts the sample size and may limit the generalizability of the results. To better assess the clinical, diagnostic, and prognostic relevance of our findings, further investigations (ideally through multicenter studies) are warranted for external validation and broader applicability.

Lastly, data on laboratory investigations did not identify any significant differences between the two sub-populations, as an example, BAL lymphocytosis at diagnosis reported on 6 women and 10 men.

## Conclusions

Sarcoidosis continues to be a challenging disease considering patients' unmet need (treatment side effects and reduced quality of life) and that no treatment consensus has been reached in some special situations. Therefore, patient phenotyping is essential to harmonize several critical aspects that remain unaddressed by current guidelines, including: identifying the risk of disease recurrence, optimizing treatment dosage and duration, assessing therapeutic response, and recognizing prognostic factors associated with unfavorable outcomes. Phenotyping is an evolving process that adapts as new genetic insights, biomarkers, and disease characteristics are discovered. With the ongoing expansion of knowledge about sarcoidosis, clinical and research-oriented phenotyping is likely to offer growing value across multiple dimensions of disease management and investigation.

## Data Availability

The raw data supporting the conclusions of this article will be made available by the authors, without undue reservation.
